# Positive Association of Fibroadenomatoid Change with HER2-Negative Invasive Breast Cancer: A Co-Occurrence Study

**DOI:** 10.1371/journal.pone.0129500

**Published:** 2015-06-22

**Authors:** Yaqin Chen, Anthony Bekhash, Albert J. Kovatich, Jeffrey A. Hooke, Jianfang Liu, Leonid Kvecher, J. Leigh Fantacone-Campbell, Edith P. Mitchell, Hallgeir Rui, Richard J. Mural, Craig D. Shriver, Hai Hu

**Affiliations:** 1 Biomedical Informatics, Windber Research Institute, Windber, Pennsylvania, United States of America; 2 Clinical Breast Care Project, Henry M. Jackson Foundation for the Advancement of Military Medicine, Rockville, Maryland, United States of America; 3 Kimmel Cancer Center, Thomas Jefferson University, Philadelphia, Pennsylvania, United States of America; 4 Clinical Breast Care Project, Murtha Cancer Center, Walter Reed National Military Medical Center, Bethesda, Maryland, United States of America; Weizmann Institute of Science, ISRAEL

## Abstract

**Background:**

Risk assessment of a benign breast disease/lesion (BBD) for invasive breast cancer (IBC) is typically done through a longitudinal study. For an infrequently-reported BBD, the shortage of occurrence data alone is a limiting factor to conducting such a study. Here we present an approach based on co-occurrence analysis, to help address this issue. We focus on fibroadenomatoid change (FAC), an under-studied BBD, as our preliminary analysis has suggested its previously unknown significant co-occurrence with IBC.

**Methods:**

A cohort of 1667 female patients enrolled in the Clinical Breast Care Project was identified. A single experienced breast pathologist reviewed all pathology slides for each case and recorded all observed lesions, including FAC. Fibroadenoma (FA) was studied for comparison since FAC had been speculated to be an immature FA. FA and Fibrocystic Changes (FCC) were used for method validation since they have been comprehensively studied. Six common IBC and BBD risk/protective factors were also studied. Co-occurrence analyses were performed using logistic regression models.

**Results:**

Common risk/protective factors were associated with FA, FCC, and IBC in ways consistent with the literature in general, and they were associated with FAC, FA, and FCC in distinct patterns. Age was associated with FAC in a bell-shape curve so that middle-aged women were more likely to have FAC. We report for the first time that FAC is positively associated with IBC with odds ratio (OR) depending on BMI (OR = 6.78, 95%CI = 3.43-13.42 at BMI<25 kg/m^2^; OR = 2.13, 95%CI = 1.20-3.80 at BMI>25 kg/m^2^). This association is only significant with HER2-negative IBC subtypes.

**Conclusions:**

We conclude that FAC is a candidate risk factor for HER2-negative IBCs, and it is a distinct disease from FA. Co-occurrence analysis can be used for initial assessment of the risk for IBC from a BBD, which is vital to the study of infrequently-reported BBDs.

## Introduction

Establishment of a benign breast disease/lesion (BBD) as a risk factor for invasive breast cancer (IBC) typically requires a longitudinal case-control study [1, 2]. For an infrequently-reported BBD, it is very difficult to conduct a longitudinal study due to the shortage of reported cases. Here we explore the possibility of using a co-occurrence analysis to estimate the risk of a BBD for IBC, until longitudinal studies can be conducted. This method could provide vital information for estimating the risk for IBC from less-frequently-reported, and as a consequence under-studied, BBDs.

In this study we focus on one infrequently reported BBD, fibroadenomatoid change (FAC). FAC is characterized by a microscopic nodule resembling a fibroadenoma, but often without the typical circumscription and proliferative stroma associated with fibroadenoma. FAC is additionally characterized by elongated, compressed glands embedded in a fibrotic stroma. Although FAC has been recognized since the early 1980s [3], literature about this lesion is sparse. In the literature FAC is often defined as prominent hyperplasia of the lobules with sclerosis of the interlobular stroma presenting as a diffuse or poorly-circumscribed tumor, bearing the composite features of fibroadenoma (FA) and fibrocystic changes (FCC) [4–7]. FAC is also known as fibroadenomatous hyperplasia, sclerosing lobular hyperplasia, fibroadenomatosis, or fibroadenomatoid mastopathy [4, 5, 7], and FAC is observed across different race groups [3, 5, 7]. The mean age of FAC diagnosis is 32–33 years, which is similar to that of patients with FCC (35 years) but notably older than that of patients diagnosed with FA (26 years) [5, 7]. In the literature, there is no information on FAC’s association with IBC or with any common risk/protective factors for IBC or BBDs. The natural history of FAC remains unclear. FAC has been speculated to represent a morphological stage in the development or degeneration of FA, or to represent the intermediate stage of a growth disorder between FCC and FA related to the influence of estrogen and progesterone [4–6].

Study of FAC has been hindered by the low number of reported cases. Up to date, no longitudinal study has been reported to assess the risk for IBC from this lesion, nor has there been a published co-occurrence study of this lesion with IBC. There are, however, a small number of studies including our own work on the co-occurrence of other BBDs including FCC and FA, with in situ or invasive breast cancers [8–12]. However, these publications have generally only reported the frequencies or co-occurrence rates of these BBDs, and have not included an odds ratio (OR) estimation. The study of FAC has also been hindered by factors negatively affecting the study of BBDs in general, for example the lack of a consistent and clear-cut clinical and histopathological definition [13]. Also, pathologists may differ in their interpretation of how to apply such definitions, causing pathologist-to-pathologist variation in disease diagnosis [14]. In addition, clinical pathologists often record only the most advanced lesion for diagnosis. Less severe lesions may be omitted from the surgical pathology report; however, from a research perspective, inclusion of this information might be helpful.

Analyzing data from the Clinical Breast Care Project (CBCP) [15–17], we determine that it is possible to study FAC using the CBCP cohort. For CBCP subjects enrolled from the Walter Reed National Military Medical Center (CBCP-WR), a single, experienced breast pathologist reviews all the pathology slides from biopsies and surgically removed tissues, and records detailed pathologic information onto a master list of 131 pathologic conditions as part of the CBCP Pathology Checklist. FAC is recorded, as well as 78 other BBDs. Thus, the data from the CBCP-WR patients overcome several of the shortcomings described earlier for the study of FAC; these data provide a good opportunity for a co-occurrence study of FAC with IBC.

We seek to determine whether co-occurrence analysis can be used for initial analysis of a BBD as risk factor for IBC. While focusing on FAC, we also study FA and FCC using the same methods and compare the findings with the literature for validation of the method. FA and FCC are selected because they have been comprehensively studied with established risk associations with IBC [13, 18–28]. We first assess common risk/protective factors for their associations with the BBDs, then study these BBDs adjusted for common risk/protective factors for their associations with IBCs and IBC subtypes. Logistic regression models are developed, and several potentially important observations are made.

## Methods

### Ethics Statement

This study was conducted in accordance with a minimal risk protocol entitled “Tissue and Blood Library Establishment for Molecular, Biochemical and Histologic Study of Breast Disease”, approved by the IRB of the Walter Reed National Military Medical Center (IRBNet #20704) for the Clinical Breast Care Project (CBCP). Written informed consent was given by participants for use of their medical records and additional data collected through the CBCP questionnaires and data forms. The consent also allowed biospecimen procurement and subsequent use for genomic and proteomic experiments. Research using these molecular and clinicopathologic data was covered by the protocol, including this specific study reported here as part of the CBCP.

### Subjects

**Fig 1:**
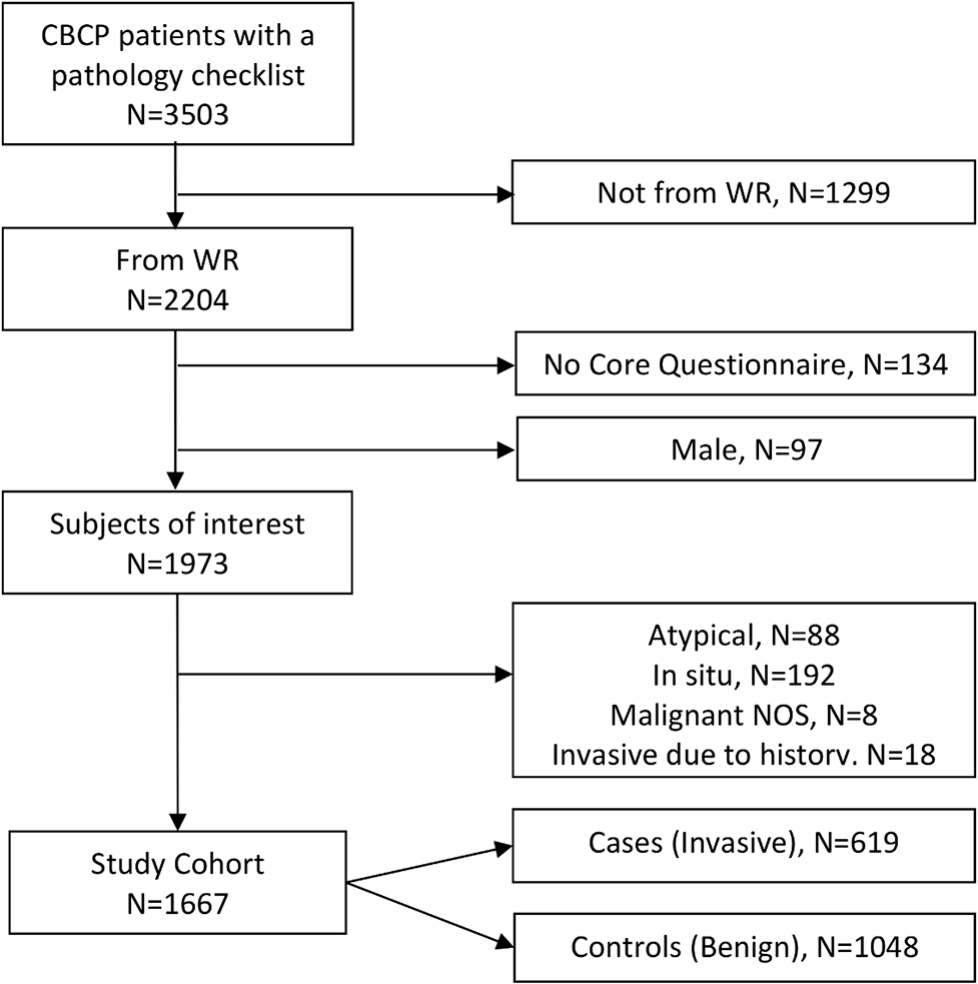
Determination of the cohort for this study. Abbreviations: CBCP, Clinical Breast Care Project; WR, Walter Reed National Military Medical Center; Malignant NOS, Malignant not otherwise specified.

This study drew from a pool of patients enrolled in the CBCP from the Walter Reed National Military Medical Center. Patients seen at the CBCP-WR clinic were military beneficiaries referred by a primary care doctor. Conditions resulting in such a referral included, an abnormal mammogram reading, a high risk family history, or other breast-related indications. A Core Questionnaire was completed for every enrolled subject by a nurse case manager, covering demographics, medical history, and risk factor information, etc. For patients undergoing a biopsy/surgery, a Pathology Checklist was completed by the CBCP pathologist to record any of 131 breast pathology lesions observed, including FAC and 78 other BBDs. A diagnosis category was assigned to each patient based on the most severe diagnosis present (i.e., Benign, Atypical, In Situ, Invasive, Other Malignant). CBCP patients enrolled between December 2000 and February 2011, who underwent a biopsy, were identified for this study, and the cohort selection was done as illustrated in Fig 1.

### Immunohistochemistry

Immunohistochemical assays for Estrogen Receptor (ER), Progesterone Receptor (PR), Human Epidermal Growth Factor Receptor 2 (HER2), and Ki67 were performed on IBC tumors by a centralized CLIA-certified laboratory following standardized protocols. The results were used for IBC subtyping [29]. A tumor was considered ER or PR positive if the corresponding nuclear staining was >5% by institutional standards at the time. The HER2 result was considered negative if IHC = 0 or 1+, positive if IHC = 3+; for IHC = 2+, Florescence In-Situ Hybridization (FISH) was used to determine the final HER2 status. Ki67 was positive if nuclear staining was ≥15%. For IBC subtypes, Luminal A (LA) = ER+/HER2-/Ki67-; Luminal B-HER2 negative (LB-HER2-) = ER+/HER2-/Ki67+; Luminal B-HER2 positive (LB-HER2+) = ER+/HER2+; HER2 Positive (HER2+) = ER-/PR-/HER2+; Triple Negative (TN) = ER-/PR-/HER2-.

### Variables of interest

We studied three BBDs, FAC, FA, and FCC, from the 131 breast pathology conditions. The CBCP pathologist considered FCC as composed of any one or combination of 4 components: stromal fibrosis, cysts, apocrine metaplasia, and sclerosing adenosis. This definition of FCC was therefore used in the CBCP program. We understood that the definition of FCC was not static and had evolved over time [30–32].

Six common risk/protective factors for IBCs and BBDs were selected from the literature; they were: 1) Current use of oral contraceptive (OC) with a binary value of Yes/No; 2) Number of live births binned into three levels of 0, 1–2, and ≥3; 3) Body mass index (BMI), binary binned into <25 kg/m^2^ or ≥25 kg/m^2^; 4) Hormonal replacement therapy (HRT), with values of Never, Estrogen only, Combo, and Other/Unknown; 5) Age, binned into <41, 41–60, and >60 years old; 6) Race, binned into African American (AA), Caucasian American (CA), Asian, and Other.

### Statistical modeling

All statistical tests were two-sided; p<0.05 was considered statistically significant. All analyses were carried out using SAS version 9.3. For logistic regression analysis, ORs and 95% confidence intervals (CIs) were calculated. The lowest level of each variable was used for reference as needed.

Multivariable logistic regression models were developed to study the association between the outcome and the variables (predictors), which was preceded by a univariate analysis for variable selection where only the variables satisfying p<0.25 qualified for modeling. A preliminary model was first built, using a stepwise selection procedure. Next, all possible two-way interactions between variables in the preliminary model were examined, using backward selection (p< 0.05 for inclusion).

Three BBD models were developed to study the association of the common risk/protective factors with FAC, FA, and FCC, respectively. For each model the BBD outcome was “Yes” or “No”, and the common risk/protective factors were used as predictors. To study the association of the three BBDs with IBCs, one IBC model was developed where the outcome was IBC Case (patient diagnosis category: Invasive) or Control (patient diagnosis category: Benign). The three BBDs, as well as the 6 common risk/protective factors, were used as the predictors. To study the association of the three BBDs with IBC subtypes, two IBC subtype models were developed, using the subtypes in reference to Control or the LA subtype as the outcome respectively. The 3 BBDs and the 6 risk/protective factors were used as the predictors.

## Results

**Fig 2:**
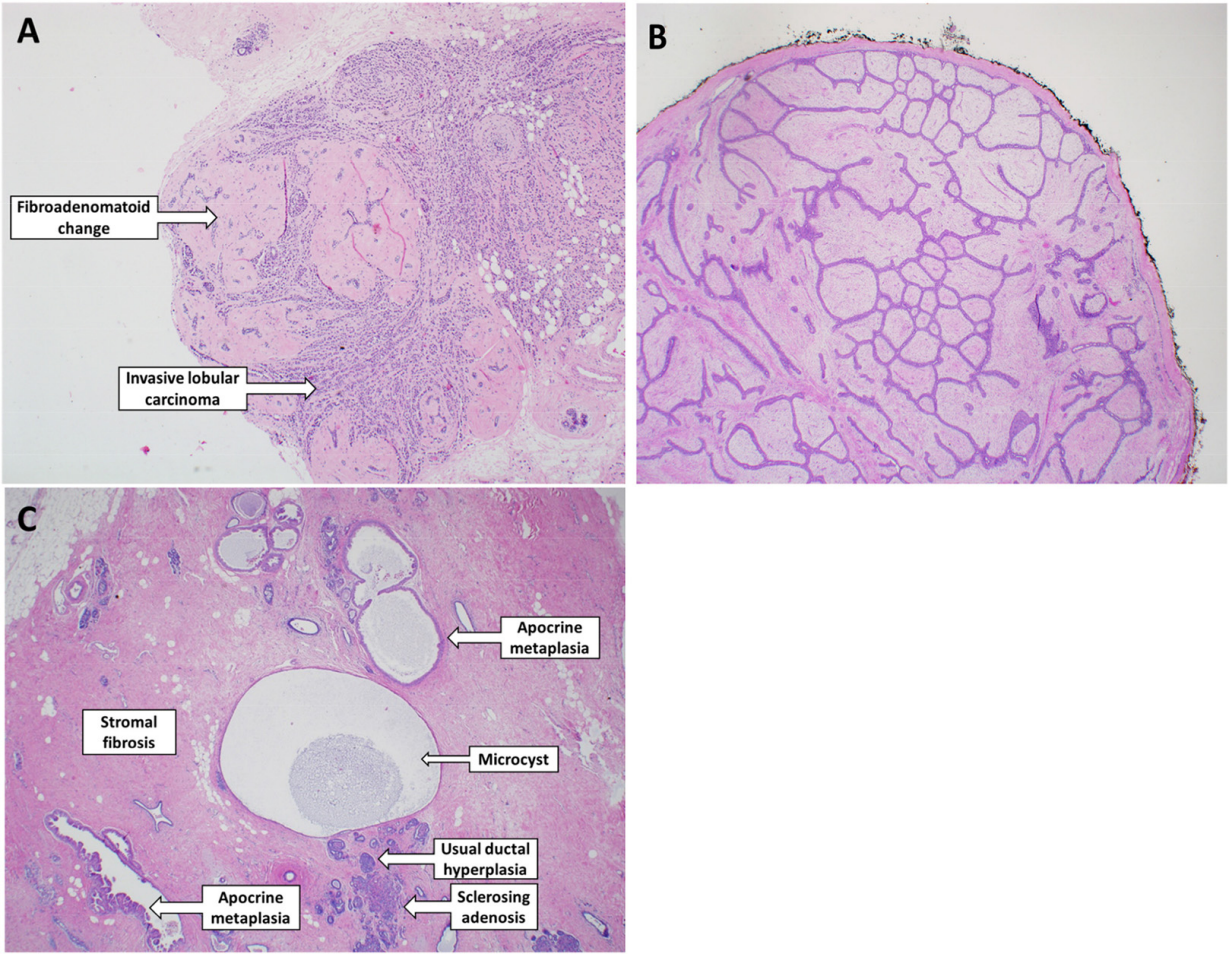
Histological images of FAC, FA, and FCC. Original magnification: 200x. **(A)** FAC. Multiple miniature fibroadenoma-like nodules are intimately associated with an invasive lobular carcinoma. Unlike an FA, the lesion is microscopic and not well-defined. **(B)** A portion of a typical FA. The lesion is well-circumscribed, has a fibrous capsule, and displays proliferation of both glandular and stromal elements. The elongated, branching epithelial glands are characteristic of FA. **(C)** FCC. This section of breast tissue exhibits many elements of FCC including stromal fibrosis, microcysts, apocrine metaplasia, sclerosing adenosis, and usual intraductal hyperplasia. The patient had ductal carcinoma *in situ* on other tissue sections.

From a total of 3503 CBCP subjects with a pathology checklist, 1667 were eligible for this study (Fig 1). There were 619 cases with an IBC, and 1048 controls with benign disease. Representative pathologic images of FAC, FA, and FCC are shown in Fig 2.

The study cohort properties are shown in Tables 1 and 2. Table 1 characterizes the three BBD cases/controls in relation to the 6 common risk/protective factors, where a BBD case is a patient positive for the BBD, and a BBD control is a patient negative for that BBD. It was observed that for both FA and FCC all 6 risk/protective factors distributed significantly differently between BBD cases and controls; yet for FAC, only age, current OC use, and the number of live births showed significance. Table 2 characterizes the IBC Cases and Controls (Benign) in relation to the 3 BBDs and the 6 factors, showing how these three BBDs co-occurr with IBCs. Here all these variables distributed significantly differently between IBC Cases and Controls. The three BBDs also co-occurred with each other and such co-occurrences are detailed in S1 Table.

**Table 1:** Characteristics of BBD cases/controls and risk/protective factors.

		FA			FAC			FCC		
		NegativeNum. (%)	PositiveNum. (%)	P-value	Negative Num. (%)	Positive Num. (%)	P-value	Negative Num. (%)	Positive Num.(%)	P-value
Total^1^		1262(100)	308(100)		1427(100)	143(100)		695(100)	875(100)	
**Age**	<41	261(21)	171(56)	< .0001	409(29)	23(16)	0.002	264(38)	168(19)	< .0001
	[41,60]	621(49)	107(35)		645(45)	83(58)		265(38)	463(53)	
	>60	380(30)	30(10)		373(26)	37(26)		166(24)	244(28)	
**Race**	AA	319(25)	102(33)	0.002	380(27)	41(29)	0.101	239(34)	182(21)	< .0001
	CA	818(65)	163(53)		901(63)	80(56)		385(55)	596(68)	
	Asian	48(4)	16(5)		58(4)	6(4)		26(4)	38(4)	
	Other	77(6)	27(9)		88(6)	16(11)		45(6)	59(7)	
**Current OC use**	No	1189(94)	262(85)	< .0001	1311(92)	140(98)	0.007⌂	613(88)	838(96)	< .0001
	Yes	73(6)	46(15)		116(8)	3(2)		82(12)	37(4)	
**Number of live birth**	0	234(19)	120(39)	< .0001	330(23)	24(17)	0.013	177(25)	177(20)	0.011
	1–2	645(51)	146(47)		725(51)	66(46)		351(51)	440(50)	
	≥3	383(30)	42(14)		372(26)	53(37)		167(24)	258(29)	
**BMI**	<25	576(46)	180(58)	< .0001	691(48)	65(45)	0.498	307(44)	449(51)	0.005
	≥25	686(54)	128(42)		736(52)	78(55)		388(56)	426(49)	
**HRT**	Never	814(65)	267(87)	< .0001	989(69)	92(64)	0.174⌂	514(74)	567(65)	< .0001
	Estrogen	181(14)	16(5)		176(12)	21(15)		84(12)	113(13)	
	Combo	230(18)	21(7)		222(16)	29(20)		79(11)	172(20)	
	Other^2^	37(3)	4(1)		40(3)	1(1)		18(3)	23(3)	

Note

^1^ cases with missing values in all 6 factors were not included

^2^ “HRT, Other” was not used for FAC model selection due to low count.

⌂ indicates P-values from Fisher exact test; all other P-values were from Chi-square test. Abbreviations: FA = Fibroadenoma; FAC = Fibroadenomatoid Change; FCC = Fibrocystic Changes; Num. = Number; Race, AA = African American, CA = Caucasian American; Current OC use = Current oral contraceptive use; BMI = Body Mass Index; HRT = Hormonal replacement therapy; Combo = Estrogen & Progesterone.

**Table 2:** Characteristics of IBC cases/controls in relationship to BBDs and risk/protective factors.

		IBC				
BBDs and other factors		Controls		Cases		P-value
Total		1048		619		
		Num.	%	Num.	%	
**FAC**						<0.0001
	No	1000	95.42	532	85.95	
	Yes	48	4.58	87	14.05	
**FA**						<0.0001
	No	741	70.71	583	94.18	
	Yes	307	29.29	36	5.82	
**FCC**						<0.0001
	No	560	53.44	237	38.29	
	Yes	488	46.56	382	61.71	
**Age**						<0.0001
	<41	434	41.41	45	7.27	
	[41,60]	463	44.18	293	47.33	
	>60	151	14.41	281	45.40	
**Race**						<0.0001
	AA	324	30.92	137	22.13	
	CA	596	56.87	426	68.82	
	Asian	44	4.20	20	3.23	
	Other	71	6.77	32	5.17	
	UN*	13	1.24	4	0.65	
**Current OC use**						<0.0001
	No	770	73.47	499	80.61	
	Yes	101	9.64	21	3.39	
	UN*	177	16.89	99	15.99	
**Number of live birth**						<0.0001
	0	267	25.48	89	14.38	
	1–2	493	47.04	296	47.82	
	≥3	227	21.66	214	34.57	
	UN*	61	5.82	20	3.23	
**BMI**						<0.0001
	<25	501	47.81	229	37.00	
	≥25	469	44.75	366	59.12	
	UN*	78	7.44	24	3.88	
**HRT**						<0.0001
	Never	763	72.81	353	57.03	
	Estrogen	108	10.31	94	15.19	
	Combo	103	9.83	124	20.03	
	Other	27	2.58	22	3.55	
	UN*	47	4.48	26	4.20	

*UN, Unknown, not used in Chi-square test.

Abbreviations: IBC = Invasive Breast Cancer; BBDs = Benign Breast Diseases; FA = Fibroadenoma; FAC = Fibroadenomatoid Change; FCC = Fibrocystic Changes; Num. = Number; Race, AA = African American, CA = Caucasian American; Current OC use = Current oral contraceptive use; BMI = Body Mass Index; HRT = Hormonal replacement therapy; Combo = Estrogen & Progesterone.

The results from the BBD model for FAC are shown in Table 3. Of all the 6 factors analyzed, only age was significantly and positively associated with FAC (p = 0.015). Number of live births (p = 0.095), race (p = 0.096) and current OC use (p = 0.077) trended towards association with FAC. No significant interaction was identified. Compared to younger women, middle-aged women were more likely to have FAC (OR = 2.029, 95% CI = 1.232–3.344).

**Table 3:** FAC and associated factors

.Effect	Odds Ratio	95% CI		P-value
**Age**				0.015
Age>60 vs <41	1.483	0.834	2.638	0.179
Age[41,60] vs <41	2.029	1.232	3.344	0.006
Age>60 vs [41,60]	0.731	0.480	1.113	0.144
**Race**				0.096
Asian vs AA	0.988	0.397	2.460	0.980
CA vs AA	0.798	0.532	1.196	0.274
Asian vs CA	1.239	0.512	2.998	0.635
**Number of live birth**				0.095
Live birth 1–2 vs 0	1.009	0.613	1.661	0.972
Live birth ≥3 vs 0	1.527	0.898	2.598	0.118
Live birth ≥3 vs 1–2	1.514	1.019	2.249	0.040
**Current OC use** Y vs N	0.344	0.105	1.125	0.077

Abbreviations: FAC = Fibroadenomatoid Change; CI = Confidence Interval; AA = African American; CA = Caucasian American; Current OC use = Current oral contraceptive use; Y = Yes; N = No.

The results from the FA model are shown in Table 4. Number of live births (p<0.0001) and age (p<0.0001) were significantly associated with FA. The interaction between age and number of live births was also significant (p = 0.006). Specifically, significant associations were detected for younger women (<41 years) but not for other age groups, and the number of live births was negatively associated with FA. There was a trend that HRT use, combination or estrogen-only, was negatively associated with FA (p = 0.102).

**Table 4:** FA and associated factors.

Effect	Odds Ratio	95% CI		P-value
**Number of live birth**				< .0001
**Age**				< .0001
Age* Number of live birth				0.006
Live birth 0 vs 1–2 at age<41	2.667	1.749	4.066	< .0001
Live birth 0 vs ≥3 at age<41	5.891	2.795	12.415	< .0001
Live birth 1–2 vs ≥3 at age<41	2.209	1.049	4.651	0.037
Age>60 vs [41,60] at Live birth = 0	0.988	0.360	2.714	0.981
Age>60 vs <41 at Live birth = 0	0.139	0.054	0.354	< .0001
Age[41,60] vs <41 at Live birth = 0	0.140	0.077	0.255	< .0001
Age>60 vs [41,60] at Live birth = 1–2	0.609	0.329	1.126	0.114
Age>60 vs <41 at Live birth = 1–2	0.283	0.147	0.547	0.0002
Age[41,60] vs <41 at Live birth = 1–2	0.465	0.310	0.698	0.0002
Age>60 vs [41,60] at Live birth > = 3	0.446	0.198	1.008	0.052
Age>60 vs <41 at Live birth > = 3	0.325	0.122	0.865	0.024
Age[41,60] vs <41 at Live birth > = 3	0.727	0.322	1.640	0.443
**HRT**				0.102
Combo vs Never	0.607	0.361	1.019	0.059
Estrogen vs Never	0.589	0.331	1.046	0.071
Combo vs Estrogen	1.030	0.520	2.043	0.933

Abbreviations: FA = Fibroadenoma; CI = Confidence Interval; HRT = Hormonal replacement therapy; Combo = Estrogen & Progesterone.

Table 5 shows modeling results for FCC. Age (p<0.0001), race (p<0.0001), current OC use (p<0.0001), and BMI (p<0.0001) were significantly associated with FCC. Compared to younger women, older and middle-aged women were at higher risk of developing FCC, and older women trended towards a lower risk of developing FCC when compared to middle-aged women. Regarding race, AA women were at a lower risk, compared to CA women. The association of current OC use with FCC depended on BMI as demonstrated by the significant interaction between the two variables (p = 0.043). In women with a BMI<25 kg/m^2^, current OC use negatively associated with FCC. On the other hand, in women not currently using OC, those with a BMI≥25 kg/m^2^ had a lower risk for FCC.

**Table 5:** FCC and associated factors.

Effect	Odds Ratio	95% CI		P-value
**Age**				< .0001
>60 vs [41,60]	0.799	0.619	1.032	0.085
>60 vs <41	2.004	1.485	2.703	< .0001
[41,60] vs <41	2.507	1.929	3.257	< .0001
**Race**				< .0001
AA vs Asian	0.653	0.373	1.141	0.135
AA vs CA	0.540	0.424	0.689	< .0001
Asian vs CA	0.827	0.485	1.413	0.488
**BMI**				< .0001
**Current OC use**				< .0001
Current OC use*BMI				0.043
Current OC use Y vs N at BMI<25	0.309	0.182	0.525	< .0001
Current OC use Y vs N at BMI≥25	0.779	0.375	1.615	0.501
BMI≥25 vs <25 at current OC use = N	0.625	0.499	0.782	< .0001
BMI≥25 vs <25 at current OC use = Y	1.572	0.659	3.751	0.308

Abbreviations: FCC = Fibrocystic Changes; CI = Confidence Interval; AA = African American, CA = Caucasian American; Current OC use = Current oral contraceptive use; BMI = Body Mass Index; Y = Yes; N = No.

Next, we developed an IBC model to study IBC association with concurrent BBDs. Of the 6 common risk/protective factors included in the model, only age and race were independently associated with IBC. The associations of BMI and HRT with IBCs were reflected as interactions with FAC or FA respectively (S2 Table). Table 6 shows the main results. Controlled for other factors, concurrent FAC positively associated with IBC (p<0.0001), so did its interaction with BMI (p = 0.011). Specifically, for women with a BMI≥25 kg/m^2^, FAC was positively associated with IBC (OR = 2.132, 95%CI = 1.197–3.796), yet for women with a BMI<25 kg/m^2^, this association was considerably stronger (OR = 6.784, 95%CI = 3.430–13.421). The model also showed that concurrent FA negatively associated with IBC (p<0.0001), and such association depended on whether the patient received HRT or not (p = 0.039). For women who had never received HRT, FA negatively associated with IBC and this association was more profound in patients not having concurrent FCC (OR = 0.334, 95%CI = 0.184–0.605 for FCC = Yes; OR = 0.104, 95%CI = 0.046–0.237 for FCC = No). For HRT receivers, only for those receiving estrogen HRT who did not have concurrent FCC, was FA negatively associated with IBC (OR = 0.120, 95%CI = 0.025–0.571). Finally, concurrent FCC alone were not associated with IBC (p = 0.184). However, the interaction FA*FCC was associated with IBC (p = 0.014). Specifically, only for women with FA were concurrent FCC positively associated with IBC (OR = 3.831, 95%CI = 1.566–9.374).

**Table 6:** Co-occurrence of BBDS with IBCs.

Effect	Odds Ratio	95% CI		P-value
**FAC**				< .0001
FAC*BMI				0.011
Y vs N at BMI<25	6.784	3.430	13.421	< .0001
Y vs N at BMI≥25	2.132	1.197	3.796	0.010
**FA**				< .0001
FA*HRT				0.039
FA:Y vs N at FCC = Y HRT = Never	0.334	0.184	0.605	0.0003
FA:Y vs N at FCC = N HRT = Never	0.104	0.046	0.237	< .0001
FA:Y vs N at FCC = N HRT = Estrogen	0.120	0.025	0.571	0.008
**FCC**				0.184
FCC*FA				0.014
FCC: Y vs N at FA = Y	3.831	1.566	9.374	0.003
FCC: Y vs N at FA = N	1.200	0.917	1.569	0.183

Abbreviations: BBD = Benign Breast Disease; IBC = Invasive Breast Cancer; CI = Confidence Interval; FA = Fibroadenoma; FAC = Fibroadenomatoid Change; FCC = Fibrocystic Changes; BMI = Body Mass Index; HRT = Hormonal replacement therapy; Y = Yes; N = No.

The observation that concurrent FAC was positively associated with IBC led us to develop a model to study its association with IBC subtypes in reference to Control (Benign). In this model, age (p = 0.0005), race (p = 0.023), and BMI (p = 0.049) were significantly associated with IBC subtypes (see S3 Table). FAC positively associated with the LA (OR = 7.220, 95%CI = 4.053–12.862), LB-HER2- (OR = 3.757, 95%CI = 1.713–8.240), and TN (OR = 5.219, 95%CI = 2.676–10.178) subtypes, but not with the LB-HER2+ nor HER2+ subtypes (Table 7). On the contrary, FA negatively associated with all the subtypes. FCC did not associate with any subtypes. Again, no other significant association or interaction was detected.

**Table 7:** Differential co-occurrence of BBDs with IBC subtypes in reference to Control.

Effect	Subtype	Odds Ratio	95% CI		P-value
**FAC**					< .0001
Y vs N	LA	7.220	4.053	12.862	< .0001
	LB-HER2-	3.757	1.713	8.240	0.001
	LB-HER2+	1.321	0.301	5.791	0.712
	HER2+	2.287	0.750	6.971	0.146
	TN	5.219	2.676	10.178	< .0001
**FA**					< .0001
Y vs N	LA	0.347	0.183	0.660	0.001
	LB-HER2-	0.339	0.142	0.807	0.015
	LB-HER2+	0.176	0.041	0.751	0.019
	HER2+	0.274	0.082	0.910	0.035
	TN	0.174	0.062	0.486	0.001
**FCC**					0.470
Y vs N	LA	1.521	1.032	2.241	0.034
	LB-HER2-	1.015	0.621	1.658	0.954
	LB-HER2+	1.050	0.517	2.131	0.893
	HER2+	0.959	0.499	1.842	0.899
	TN	1.021	0.634	1.644	0.933

Abbreviations: BBD = Benign Breast Disease; IBC = Invasive Breast Cancer; CI = Confidence Interval; FAC = Fibroadenomatoid Change; FA = Fibroadenoma; FCC = Fibrocystic Changes; LA = Luminal A subtype; LB-HER2- = Luminal B-HER2 negative subtype; LB-HER2+ = Luminal B-HER2 positive subtype; HER2+ = HER2 positive subtype; TN = Triple Negative subtype. Y = Yes; N = No.

We further investigated whether FAC preferentially associated with any of the three subtypes lacking expression of HER2. Thus a similar IBC subtype model was developed, in reference to LA this time. Again age (p<0.001), race (p = 0.023), and BMI (p = 0.004) were significantly associated with IBC subtypes (see S4 Table). Here again, only FAC (p = 0.033), but not FA or FCC, was significantly associated with IBC subtypes in reference to LA (Table 8). FAC was not associated with LB-HER2- or TN subtypes relative to LA, but it was negatively associated with the HER2+ subtype (OR = 0.246, 95%CI = 0.08–0.80) and the LB-HER2+ subtype (OR = 0.151, 95%CI = 0.032–0.711) relative to LA.

**Table 8:** Differential co-occurrence of BBDs with IBC subtypes in reference to LA.

Effect	Subtype	Odds Ratio	95% CI		P-value
**FAC**					0.033
Y vs N	LB-HER2-	0.543	0.238	1.240	0.147
	LB-HER2+	0.151	0.032	0.711	0.017
	HER2+	0.246	0.075	0.800	0.020
	TN	0.570	0.269	1.207	0.142
**FA**					0.894
Y vs N	LB-HER2-	0.827	0.161	4.233	0.8194
	LB-HER2+	1.336	0.459	3.887	0.5955
	Her2+	1.090	0.277	4.281	0.9019
	TN	0.670	0.199	2.254	0.5181
**FCC**					0.249
Y vs N	LB-HER2-	0.649	0.366	1.153	0.140
	LB-HER2+	0.576	0.256	1.299	0.184
	HER2+	0.589	0.284	1.223	0.155
	TN	0.561	0.315	0.999	0.050

Abbreviations: BBD = Benign Breast Disease; IBC = Invasive Breast Cancer; CI = Confidence Interval; FAC = Fibroadenomatoid Change; FA = Fibroadenoma; FCC = Fibrocystic Changes; LA = Luminal A subtype; LB-HER2- = Luminal B-HER2 negative subtype; LB-HER2+ = Luminal B-HER2 positive subtype; HER2+ = HER2 positive subtype; TN = Triple Negative subtype. Y = Yes; N = No.

## Discussion

FAC, FA, and FCC are associated with the 6 common risk/protective factors in distinct patterns. FAC only significantly associated with age (Table 3), exhibiting a bell-shaped risk distribution such that middle-aged women had the highest risk compared to younger women (a significant OR = 2.029) and older women (a trend OR = 1.368, reciprocal of OR = 0.731 for older women vs. middle-aged women). Race trended towards association with FAC, as did the number of live births (positive trend), and current OC use (negative trend). FA, on the other hand, negatively associated with age and the number of live births (Table 4). The interaction Age*Number of live births indicated that for younger women, FA negatively associated with the number of live births. Thus, despite the controversy in the literature in regard to full-term pregnancies being risk or “protective” factors for FA [24, 25, 33], our results support full-term pregnancies as being a protective factor. Our study also confirms that younger women are more likely to be diagnosed with FA [13, 23]. In addition, FA trended towards a negative association with the use of HRT (Estrogen only or Combination), and this observation is also consistent with the literature that ERT (HRT-Estrogen) use in general does not result in increased risk for FA [13, 25]. In this study, an FA case was defined as a subject with an FA lesion whether it was a simple or complex FA, as we wished to use the same analysis method across the board for all the three BBDs. We also point out that although BMI did not make to the final model for FA, in the initial univariate analysis BMI was significantly and negatively associated with FA (OR = 0.597, CI = 0.464–0.768, and p<0.0001). While this finding will need to be independently verified, we caution against the validity of the consensus “protective” effect of obesity on FA reported in the literature [23, 24, 33]. We report for the first time that FCC has a negative association with current OC use in women with a BMI<25 kg/m^2^ (OR = 0.309) but not in women with a BMI≥25 kg/m^2^ (Table 5). For women who did not have current OC use, BMI was significantly and negatively associated with FCC (OR = 0.625), and this result is consistent with literature that found obesity to be “protective” for FCC [23, 24]. FCC also associated with age and race as reported in the literature [13, 23].

By developing logistic regression models for IBC prediction, we report here for the first time that FAC is positively associated with IBC (Table 6). This association is significant for women with a BMI≥25 kg/m^2^ (OR = 2.132), and is even more profound for women with a BMI<25 kg/m^2^ (OR = 6.784). While these observations will need to be independently validated, their reliability is supported by the results shown in S2 Table from the same model. These results show that common risk factors associated with IBC are consistent with the consensus in the literature for age, race, and BMI [34–38]. Specifically regarding BMI, women without FAC had a higher BMI positively associated with IBC (OR = 1.495), whereas women with FAC had a higher BMI negatively associated with IBC (OR = 0.470). Realizing that only 4.58% of Controls and 14.05% of Cases are positive for FAC (Table 2), and the fact that most of the subjects in this study are post-menopausal (data not shown but were partially reflected in age distribution in Table 2), the observed association of BMI with IBC is consistent with the literature that found increased BMI is a risk for IBC in post-menopausal women [37, 38].

It is intriguing that FAC associates significantly only with IBC HER2-negative subtypes (OR = 7.220 for LA, 3.757 for LB-HER2-, and 5.219 for TN), see Table 7. Moreover, FAC association with the LA subtype is comparable to its associations with the LB-HER2- and TN subtypes (Table 8). Such selective associations bring up an interesting question in regard to whether the HER2 signaling pathway prohibits the co-occurrence of FAC with IBC. It will also be interesting to see whether FAC is associated with treatment outcome.

Besides producing new observations related to FAC, these logistic models also enabled us to study the associations of IBC with two other BBDs, FA and FCC, whose risks for IBC development have been widely reported [13, 18–28]. Although not without controversy, the general consensus has been that simple FA is not a risk for IBC but complex FA is; non-proliferative FCC are not a risk but proliferative FCC are. Our analyses indicate that FA is negatively associated with IBC and that FCC are not associated with IBC (Table 6); these results are consistent with many studies (e.g. [8, 39]). The negative association of FA with IBC is reflected in the significant interaction FA*HRT, with ORs ranging from 0.104 to 0.334 depending on the status of FCC and whether only estrogen HRT was used or HRT was never used. Such detailed association relationships have not been reported before. We notice that the IBC association with the interaction FA*HRT depends on FCC status, which seemed to suggest a 3-way interaction. To be prudent we re-analyzed the data by including all 3-way interactions to this model, but found out that none of them were significant. Thus this nominal “3-way interaction” was introduced by the significant interaction FCC*FA (see below) to the interaction FA*HRT bridged by FA.

Our model also showed that the interaction FCC*FA is significantly associated with IBC, which has also previously not been explicitly reported. FCC positively associated with IBC only when there was co-occurring FA. We believe that this interaction reflects the reported results that complex FA has a slightly increased risk for breast cancer [19, 21, 22], because three of the four lesions that constitute FCC (cysts, apocrine metaplasia and sclerosing adenosis) also constitute complex FA [19, 32]. Thus, the results from our models may help explain some of the conflicting reports in the literature on whether FCC or FA is a risk factor for IBC.

Our study provides new observations to the literature on BBD association with IBC subtypes. Unlike FAC, the negative association of FA with IBC was uniform across IBC subtypes, and the lack of association of FCC with IBC was also uniform across all subtypes. Thus the selective association with IBC subtypes is unique for FAC, which enhances our confidence in the reliability of the results from this co-occurrence study.

While we are encouraged by the findings from this study, we recognize the differences between this co-occurrence analysis and a longitudinal study. Under several circumstances where a BBD is associated with IBC, a co-occurrence analysis could suggest that the BBD is a risk factor for IBC: 1) the co-occurring BBD is a precursor of IBC and the BBD persists through the development of the IBC; 2) the co-occurring BBD and the IBC are initiated from a common mutational ancestor; 3) a proliferative environment that facilitates IBC development also favors development of the co-occurring BBD. A positive association of a BBD with IBC through the co-occurrence analysis warrants a longitudinal study to validate whether this BBD is indeed a risk factor for IBC. Mutational and genomic analyses of IBC and co-occurring BBD need to be done to determine whether a BBD is in fact a precursor lesion to the IBC and not simply an associated lesion.

Another factor we consider is that the CBCP-WR cohort is a breast disease patient cohort. Thus, the odds of detecting FAC are higher compared to observations from the general population. However, the elevations of the odds in both IBC and control groups should be similar, and consequently should cancel each other out when deriving the OR reported in this co-occurrence study for the association of FAC with IBC. The OR trend direction (>1 or <1) should hold compared to what would be observed from the general population.

In summary using co-occurrence analyses, we report for the first time that FAC is positively associated with IBC. Our subtype co-occurrence model extends this association to only HER2-negative IBC subtypes. These results warrant a longitudinal study to determine whether FAC is indeed a risk factor for IBC. The associations from these analyses for FA and FCC with IBC are in general consistent with the literature, and we also make new findings that help to explain some of the controversial reports on risks for IBC from FA and FCC. These observations suggest that co-occurrence analysis can be used for the initial assessment of risks for IBC from BBDs. We further report that FA is negatively associated with IBC, and that common risk/protective factors associate with FAC and FA in distinct patterns.

We conclude that FAC is positively associated with IBC, and furthermore only with HER2-negative subtypes. In addition, FAC is most likely a distinct lesion from FA and should not be considered as an immature form of FA. Our results also suggest that co-occurrence analysis can be used for initial assessment of risks for IBC from BBDs, and this conclusion is important to the study of less-frequently reported BBDs for which conducting a longitudinal risk assessment for IBC is difficult.

## Supporting Information

S1 Table Concurrence of FAC, FA, and FCC lesions. (DOCX)

S2 Table Association of risk factors to IBCs (Supplemental to Table 6). (DOCX)

S3 Table Differential association of risk factors with IBC subtypes in reference to Control (Supplemental to Table 7). (DOCX)

S4 Table Differential association of risk factors with IBC subtypes in reference to LA (Supplemental to Table 8). (DOCX)
