# Supplementary material for: Positive Association of Fibroadenomatoid Change with HER2-Negative Invasive Breast Cancer: A Co-Occurrence Study
Source: PLoS One. 2015 Jun 22;10(6):e0129500. doi: 10.1371/journal.pone.0129500 (PMC4476726; doi:10.1371/journal.pone.0129500)
Supplement: S1 Table — (DOCX) [file pone.0129500.s001.docx]

**S1 Table.** Concurrence of FAC, FA, and FCC lesions

|  | **FAC** | **FA** | **FCC** | **FA*FAC*FCC** |
| --- | --- | --- | --- | --- |
| **FAC** | 147 | 18 | 122 | 16 |
| **FA** |  | 357 | 135 |  |
| **FCC** |  |  | 943 |  |

Abbreviations: FAC=Fibroadenomatoid Change;

FA=Fibroadenoma; FCC=Fibrocystic Changes
