# Supplementary material for: Positive Association of Fibroadenomatoid Change with HER2-Negative Invasive Breast Cancer: A Co-Occurrence Study
Source: PLoS One. 2015 Jun 22;10(6):e0129500. doi: 10.1371/journal.pone.0129500 (PMC4476726; doi:10.1371/journal.pone.0129500)
Supplement: S2 Table — (DOCX) [file pone.0129500.s002.docx]

**S2 Table.** Association of risk factors to IBCs (Supplemental to Table 6)

| **Effect** | **Odds Ratio** | **95% CI** | | **P-value** |
| --- | --- | --- | --- | --- |
| **Age** |  |  |  | <.0001 |
| Age >60 vs <41 | 17.901 | 11.34 | 28.26 | <.0001 |
| Age [41,60] vs <41 | 5.033 | 3.422 | 7.40 | <.0001 |
| Age >60 vs [41,60] | 3.557 | 2.616 | 4.84 | <.0001 |
| **Race** |  |  |  | 0.023 |
| AA vs CA | 0.661 | 0.492 | 0.888 | 0.006 |
| **BMI** |  |  |  | 0.004 |
| BMI ≥25 vs <25 at FAC=Y | 0.470 | 0.200 | 1.104 | 0.083 |
| BMI≥25 vs <25 at FAC=N | 1.495 | 1.140 | 1.962 | 0.004 |
| **HRT** |  |  |  | 0.007 |
| HRT Combo vs Estrogen at FA=Y | 5.836 | 1.028 | 33.143 | 0.047 |
| HRT Combo vs Never at FA=Y | 3.548 | 1.151 | 10.940 | 0.028 |
| HRT Combo vs Never at FA=N | 0.645 | 0.446 | 0.933 | 0.020 |
| HRT Estrogen vs Never at FA=N | 0.531 | 0.361 | 0.782 | 0.001 |

Abbreviations: AA = African American, CA= Caucasian American; BMI=Body Mass Index; HRT=Hormonal replacement therapy; Combo=Estrogen & Progesterone; Y=Yes; N=No.
