# Supplementary material for: Positive Association of Fibroadenomatoid Change with HER2-Negative Invasive Breast Cancer: A Co-Occurrence Study
Source: PLoS One. 2015 Jun 22;10(6):e0129500. doi: 10.1371/journal.pone.0129500 (PMC4476726; doi:10.1371/journal.pone.0129500)
Supplement: S3 Table — (DOCX) [file pone.0129500.s003.docx]

**S3 Table.** Differential association of risk factors with IBC subtypes in reference to Control (Supplemental to Table 7)

| **Effect** | **Subtype** | **Odds Ratio** | **95% CI** | | **P-value** |
| --- | --- | --- | --- | --- | --- |
| **Age** |  |  |  |  | <.0001 |
| [41,60] vs <41 | LA | 11.654 | 4.589 | 29.594 | <.0001 |
|  | LB-HER2- | 2.504 | 1.118 | 5.608 | 0.0256 |
|  | LB-HER2+ | 1.932 | 0.828 | 4.503 | 0.1275 |
|  | HER2+ | 3.402 | 1.254 | 9.235 | 0.0162 |
|  | TN | 3.668 | 1.793 | 7.503 | 0.0004 |
| >60 vs <41 | LA | 39.462 | 15.310 | 101.719 | <.0001 |
|  | LB-HER2- | 10.819 | 4.840 | 24.184 | <.0001 |
|  | LB-HER2+ | 1.674 | 0.556 | 5.044 | 0.3599 |
|  | HER2+ | 7.289 | 2.527 | 21.028 | 0.0002 |
|  | TN | 6.778 | 3.115 | 14.752 | <.0001 |
| **Race** |  |  |  |  | 0.047 |
| CA vs AA | LA | 2.384 | 1.487 | 3.822 | 0.0003 |
|  | LB-HER2- | 1.080 | 0.638 | 1.830 | 0.7739 |
|  | LB-HER2+ | 1.521 | 0.667 | 3.469 | 0.3185 |
|  | HER2+ | 1.074 | 0.510 | 2.260 | 0.8515 |
|  | TN | 0.688 | 0.422 | 1.123 | 0.1348 |
| Asian vs AA | LA | 2.149 | 0.765 | 6.037 | 0.1464 |
|  | LB-HER2- | 0.902 | 0.191 | 4.259 | 0.8965 |
|  | LB-HER2+ | <0.001 | <0.001 | 999.999 | 0.9727 |
|  | HER2+ | 1.333 | 0.269 | 6.597 | 0.7250 |
|  | TN | 0.773 | 0.215 | 2.783 | 0.6939 |
| **BMI** |  |  |  |  | 0.008 |
| ≥25 vs <25 | LA | 1.546 | 1.052 | 2.273 | 0.0266 |
|  | LB-HER2- | 2.771 | 1.570 | 4.890 | 0.0004 |
|  | LB-HER2+ | 0.938 | 0.467 | 1.884 | 0.8571 |
|  | HER2+ | 0.985 | 0.513 | 1.888 | 0.9627 |
|  | TN | 1.221 | 0.753 | 1.981 | 0.4184 |

Abbreviations: FAC=Fibroadenomatoid Change; FA=Fibroadenoma; FCC=Fibrocystic Changes; Y=Yes; N=No; LA= Luminal A subtype; LB-HER2-= Luminal B-HER2 negative subtype; LB-HER2+=Luminal B-HER2 positive subtype; HER2+=HER2 positive subtype; TN=Triple Negative subtype. AA = African American, CA= Caucasian American;

BMI=Body Mass Index.
