# Supplementary material for: Positive Association of Fibroadenomatoid Change with HER2-Negative Invasive Breast Cancer: A Co-Occurrence Study
Source: PLoS One. 2015 Jun 22;10(6):e0129500. doi: 10.1371/journal.pone.0129500 (PMC4476726; doi:10.1371/journal.pone.0129500)
Supplement: S4 Table — (DOCX) [file pone.0129500.s004.docx]

**S4 Table.** Differential association of risk factors with IBC subtypes in reference to LA (Supplemental to Table 8)

| **Effect** | **Subtype** | **Odds Ratio** | **95% CI** | | **P-value** |
| --- | --- | --- | --- | --- | --- |
| **Age** |  |  |  |  | 0.0005 |
| >60 vs <41 | LB-HER2- | 0.254 | 0.074 | 0.869 | 0.0290 |
|  | LB-HER2+ | 0.036 | 0.008 | 0.154 | <.0001 |
|  | HER2+ | 0.159 | 0.039 | 0.652 | 0.0107 |
|  | TN | 0.169 | 0.050 | 0.567 | 0.0040 |
| [41,60] vs <41 | LB-HER2- | 0.211 | 0.061 | 0.725 | 0.0135 |
|  | LB-HER2+ | 0.140 | 0.039 | 0.504 | 0.0026 |
|  | HER2+ | 0.264 | 0.067 | 1.044 | 0.0577 |
|  | TN | 0.338 | 0.104 | 1.093 | 0.0700 |
| **Race** |  |  |  |  | 0.023 |
| Asian vs AA | LB-HER2- | 0.214 | 0.023 | 1.976 | 0.1739 |
|  | LB-HER2+ | <0.001 | <0.001 | 999.9 | 0.9819 |
|  | HER2+ | 0.575 | 0.093 | 3.548 | 0.5515 |
|  | TN | 0.333 | 0.072 | 1.545 | 0.1602 |
| CA vs AA | LB-HER2- | 0.468 | 0.242 | 0.903 | 0.0235 |
|  | LB-HER2+ | 0.686 | 0.264 | 1.779 | 0.4382 |
|  | HER2+ | 0.456 | 0.195 | 1.066 | 0.0699 |
|  | TN | 0.271 | 0.144 | 0.508 | <.0001 |
| **BMI** |  |  |  |  | 0.049 |
| ≥25 vs <25 | LB-HER2- | 1.837 | 0.962 | 3.510 | 0.0656 |
|  | LB-HER2+ | 0.594 | 0.269 | 1.312 | 0.1975 |
|  | HER2+ | 0.585 | 0.283 | 1.211 | 0.1488 |
|  | TN | 0.862 | 0.481 | 1.545 | 0.6173 |

Abbreviations: FAC=Fibroadenomatoid Change; FA=Fibroadenoma; FCC=Fibrocystic

Changes; Y=Yes; N=No; LA= Luminal A subtype; LB-HER2-= Luminal B-HER2 negative subtype; LB-HER2+=Luminal B-HER2 positive subtype; HER2+=HER2 positive subtype; TN=Triple Negative subtype. AA = African American, CA= Caucasian American; BMI=Body Mass Index.
